# MAFB and MAF Transcription Factors as Macrophage Checkpoints for COVID-19 Severity

**DOI:** 10.3389/fimmu.2020.603507

**Published:** 2020-11-18

**Authors:** Miguel A. Vega, Miriam Simón-Fuentes, Arturo González de la Aleja, Concha Nieto, María Colmenares, Cristina Herrero, Ángeles Domínguez-Soto, Ángel L. Corbí

**Affiliations:** Myeloid Cell Laboratory, Centro de Investigaciones Biológicas Margarita Salas, CSIC, Madrid, Spain

**Keywords:** macrophage, innate immunity, COVID-19, MAFB, MAF

## Abstract

Defective IFN production and exacerbated inflammatory and pro-fibrotic responses are hallmarks of SARS-CoV-2 infection in severe COVID-19. Based on these hallmarks, and considering the pivotal role of macrophages in COVID-19 pathogenesis, we hypothesize that the transcription factors MAFB and MAF critically contribute to COVID-19 progression by shaping the response of macrophages to SARS-CoV-2. Our proposal stems from the recent identification of pathogenic lung macrophage subsets in severe COVID-19, and takes into consideration the previously reported ability of MAFB to dampen IFN type I production, as well as the critical role of MAFB and MAF in the acquisition and maintenance of the transcriptional signature of M-CSF–conditioned human macrophages. Solid evidences are presented that link overexpression of MAFB and silencing of MAF expression with clinical and biological features of severe COVID-19. As a whole, we propose that a high MAFB/MAF expression ratio in lung macrophages could serve as an accurate diagnostic tool for COVID-19 progression. Indeed, reversing the macrophage MAFB/MAF expression ratio might impair the exacerbated inflammatory and profibrotic responses, and restore the defective IFN type I production, thus becoming a potential strategy to limit severity of COVID-19.

## Introduction

Severe acute respiratory syndrome coronavirus 2 (SARS-CoV-2), the etiological agent of Coronavirus Disease 2019 (COVID-19), has so far caused more than 1,000,000 deaths globally (data from the WHO), and its huge clinical and social impact continues to be rising in spite of unprecedented worldwide measures to limit its transmission and pathogenicity. Following the COVID-19 outbreak, a large amount of epidemiological, clinical, and immunological information has been increasingly gathered, as a complete understanding of COVID-19 pathogenesis should allow the design of effective measures to boost the generation of effective anti-SARS-CoV-2 immune responses. More than 80% of symptomatic COVID-19 patients present fever, cough and dyspnea (mild cases), while 14% develop acute respiratory distress syndrome (ARDS) and systemic inflammation (severe cases), and 5% show fibrosis at several organs, including lungs, and coagulopathies, all of which lead to multiorgan failure (critical COVID-19 patients) (1). An imbalanced host response to SARS-CoV-2 drives the development of COVID-19, as SARS-CoV-2 infection hampers IFN production and weakens antiviral defenses, but concomitantly promotes an exacerbated production of cytokines (“cytokine storm”) and profibrotic factors, and enhanced recruitment and accumulation of leukocytes in tissues causing ARDS (2, 3). From an immunological perspective, severe COVID-19 patients display a robust immune dysregulation, including T cell and NK cell cytopenia, sustained cytokine production and hyper-inflammation (4). These two later manifestations closely resemble the macrophage activation syndrome (5), a ﻿dysregulated macrophage response induced by infections that results in a severe damage to the host tissues. It is currently accepted that macrophages lie in the center of the COVID-19 pathogenesis, and that, upon SARS-CoV-2 infection, the excessive activation of pulmonary macrophages, besides contributing to viral control, causes lung injury through the inflammatory “cytokine storm” that spreads from the lung throughout the body *via* the systemic circulation in COVID-19 patients (6, 7). Therefore, understanding the response of monocytes and macrophages to SARS-CoV-2 infection (to the virus itself, and to the systemic alterations triggered upon SARS-CoV-2 interaction with other cell types) is essential to identify potential therapeutic targets for COVID-19.

## MAFB and MAF Transcription Factors in Macrophages

MAFB and MAF belong to the large-MAF subfamily of transcription factors that includes MAF, MAFA, MAFB, and NRL, which drive terminal differentiation in numerous cell lineages (8). Although MAFB and MAF display redundant roles in differentiation processes [e.g., epidermal differentiation (9)], they can also exert opposite functions, as in the case of cortical interneuron development (10). Within the hematopoietic lineage, MAFB is preferentially expressed in most tissue-resident macrophages, whose specific enhancers contain an overrepresentation of MAF-responsive element (MARE) sequences (11), and where it promotes macrophage differentiation (12), and inhibits stemness and self-renewal of differentiated monocytes and macrophages in cooperation with MAF (13–15). Regarding macrophage cell biology, both MAFB and MAF favor the maintenance of an “M2-like” homeostatic and reparative phenotype in macrophages (16, 17). Thus, MAFB and MAF determine the anti-inflammatory and immunosuppressive polarization of M-CSF–dependent human macrophages (16) and Tumor-Associated Macrophages (TAMs) (18, 19). Indeed, silencing of MAF expression in macrophages leads to macrophage re-programming toward an M1-like phenotype and enhancement of antitumor activities (19, 20). In addition, MAFB has the ability of negatively regulating the expression of type I IFN upon viral infection by setting a threshold for IRF3-dependent transcription (21, 22). Specifically, MAFB antagonizes antiviral responses as it blocks the recruitment of coactivators to the transcription factor IRF3 (22), and suppresses the monocyte production of type I IFN in chronic hepatitis C patients (21). This function as “rheostat” for type I IFN (23) suggests that MAFB might contribute to the defective IFN production in COVID-19.

## MAFB/MAF in COVID-19

**Figure 1:**
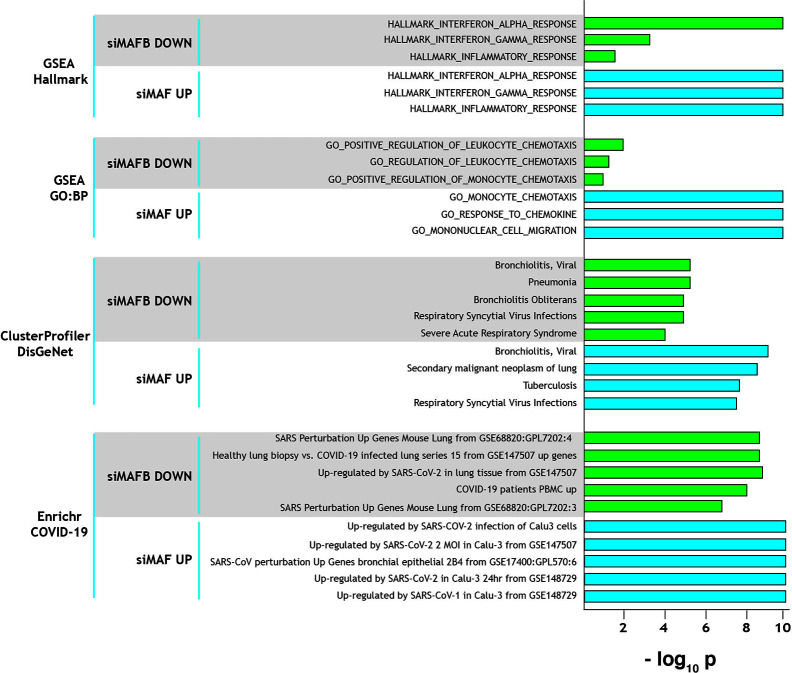
Summary of gene ontology evidences for the involvement of MAF and MAFB in COVID-19 progression and pathology-associated clinical parameters. Ontology terms significantly associated to genes downregulated by MAFB-specific siRNA (positively regulated by MAFB, siMAFB DOWN) or upregulated by MAF-specific siRNA (negatively regulated by MAF, siMAF UP), with indication of the analyzed databases. Normalized statistical significance of each association (−log_10_ p) is shown, and derived from FDRq values (for GSEA), q values (clusterProfiler) and adjusted p (Enrichr).

The role of MAFB and MAF in shaping macrophage phenotype and polarization prompted us to identify the genes regulated by these transcription factors. To this end, we analyzed the transcriptional profiles of M-CSF–conditioned human monocyte-derived macrophages (as a model of homeostatic tissue-resident macrophages) after siRNA-mediated knockdown of either MAFB (siMAFB) or MAF (siMAF) (GEO accession number: GSE155719), what led to the identification of more than 1000 genes whose expression is MAFB- and/or MAF-dependent (adjp < 0.05; |Fold change| > 1.5, GSE155719) (*Simón-Fuentes et al.*, *manuscript in preparation*). Extensive gene ontology analysis of the MAFB/MAF-dependent genes provided initial clues on the relevance of both factors in shaping the transcriptome of homeostatic/tissue-resident macrophages. First, Gene Set Enrichment Analysis (GSEA) on the Hallmark gene sets collection (24) revealed that the genes positively regulated by MAFB but inhibited by MAF are significantly enriched in terms like “HALLMARK_INTERFERON_ALPHA_RESPONSE”, “HALLMARK_INTERFERON_GAMMA_RESPONSE”, and “HALLMARK_INFLAMMATORY_RESPONSE” (**Figure 1**), which are strongly associated to the innate immune response against SARS-CoV-2 infection. This enrichment is in agreement with the involvement of MAFB and MAF in type I IFN expression (22) and the establishment of a ‘‘negative IFN-gamma signature’’ in inflammatory macrophages, respectively (17). Noteworthy, comparison of the GSEA leading edges for the “HALLMARK_INFLAMMATORY_RESPONSE” and “HALLMARK_INTERFERON_ALPHA_RESPONSE” gene sets with both factors only showed a partial overlap, implying that MAFB and MAF might affect both functions through regulation of common and specific genes (*Simón-Fuentes et al.*, *manuscript in preparation*). Regarding the “HALLMARK_INFLAMMATORY_RESPONSE” gene set, MAFB and MAF were found to oppositely regulate the expression of a cluster of chemokine-encoding genes (*CXCL10*, *CCL2*, *CCL7*, *CXCL9*) that are associated to the COVID-19 “cytokine storm” (25). A similar result was obtained upon analysis of the GO Biological Pathways gene sets sub-collection, which pointed to the involvement of the genes regulated by both factors in leukocyte migration, another COVID-19 trait (**Figure 1**). Next, analysis of the genes differentially regulated by MAFB and MAF on the disease-associated database DisGeNet using the clusterProfiler tool (26) revealed a very significant enrichment of several terms related to respiratory deterioration (a firmly established symptom of COVID-19) (**Figure 1**). In fact, Enrichr analysis (27) on the COVID-19 gene sets supported the association between MAFB- and MAF-dependent genes and COVID-19 (**Figure 1**). Further, analysis of the Wikipathways database by clusterProfiler revealed a significant association to the term COVID-19-AOP (adverse outcome pathway). Specifically, MAF knock-down was found associated to *IL7*, *CCL2*, *IL1B*, *CXCL8*, *TNF*, and *CCL3* (q value, 2.82 × 10^−4^) while MAFB was associated to *CCL2*, *IL2RA*, *IL10* and *CXCL10* expression (q value, 6.73 × 10^−3^). Altogether, these analyses strongly suggest a link between MAF/MAFB-regulated genes and COVID-19 pathology.

## MAF and MAFB Shape the Transcriptional Profile of TLR7-Activated Human Macrophages

**Figure 2:**
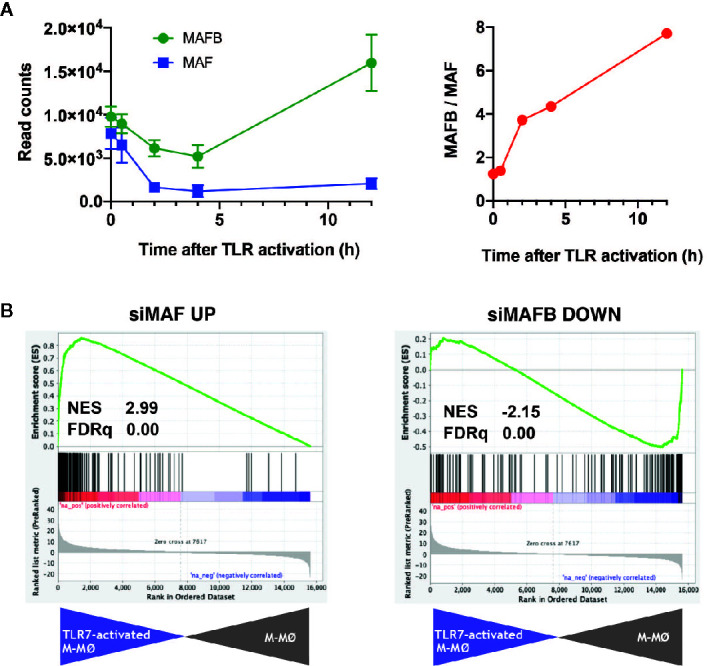
Expression of *MAF*, *MAFB* and MAF/MAFB-dependent genes along TLR7 activation of M-CSF–dependent human macrophages. **(A)** (Left panel) Expression of *MAF* and *MAFB* in M-CSF–dependent human monocyte-derived macrophages exposed to the TLR ligand CL264 (100 ng/ml) (GSE156921). (Right panel) Evolution of the *MAFB*/*MAF* expression ratio along TLR7 activation of M-CSF–dependent human monocyte-derived macrophages. **(B)** GSEA of the gene sets containing genes inhibited by MAF (siMAF UP, left panel) or upregulated by MAFB (siMAFB DOWN, right panel) on the ranked comparison of the transcriptome of TLR7-activated M-CSF–dependent human monocyte-derived macrophages (12 h, TLR7-activated M-MØ) versus the transcriptome of untreated macrophages (M-MØ). Normalized Enrichment Score (NES) and FDRq values are indicated.

TLR7 is a sensor for SARS-Cov-2, and its relevance in COVID-19 is highlighted by the discovery of the association of loss-of-function variants of X-chromosomal TLR7 with impaired type I and II IFN responses and with severe COVID-19 (28). Thus, we next assessed the expression of MAFB/MAF and their regulated genes in human M-CSF–derived macrophages exposed to the TLR7 synthetic ligand CL264 (GEO accession number: GSE156921) (*Simón-Fuentes et al.*, *manuscript in preparation*). The expression of MAFB and MAF in macrophages followed different kinetics along TLR7 activation, with a huge decrease in MAF expression and a continuously increasing MAFB/MAF expression ratio (**Figure 2A**). If this trend is maintained at longer times, the high-MAFB/low-MAF scenario would favor/promote COVID-19 pathology, as we have suggested above. Importantly, GSEA on the transcriptome of TLR7-activated macrophages (12 h) revealed a significantly positive enrichment of genes inhibited by MAF, and a concomitant negative enrichment of MAFB-regulated genes (**Figure 2B**), again supporting an opposite role for MAFB and MAF in determining the macrophage response to TLR7 activation, and hence in the response to SARS-Cov-2 infection.

## MAFB and MAF Control Gene Expression in Pulmonary Macrophages From COVID-19 Patients

**Figure 3:**
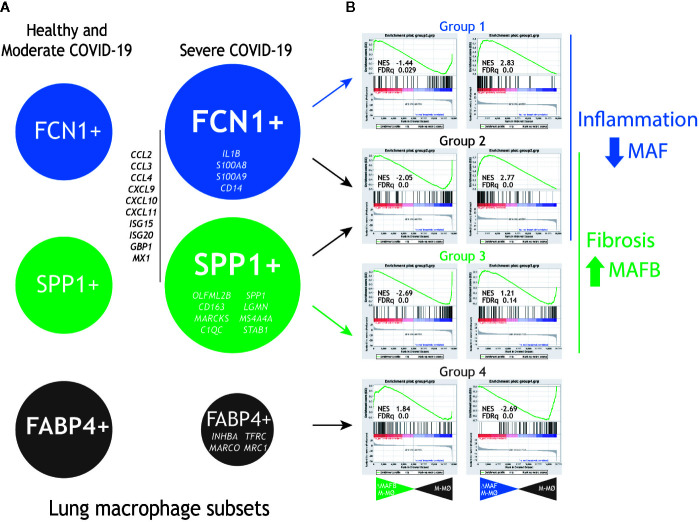
Expression of *MAF-* and *MAFB-*dependent genes in human lung macrophage subsets associated to pulmonary fibrosis and COVID-19 severity. **(A)** Previously identified human lung macrophage subsets (29–32), with indication of their relative levels in healthy individuals and moderate COVID-19 patients (left) and severe COVID-19 patients (right), as well as representative genes specifically expressed by each macrophage subset. **(B)** GSEA of the gene sets that define the human lung macrophage subsets (Group1 for the FCN1+ subset, Group 2 for the FCN1+ and SPP1+ subsets, Group 3 for the SPP1+ subset, Group 4 for the FABP4+ subset) on the ranked comparison of the transcriptomes of MAFB-specific siRNA-transfected (siRNA MAFB M-MØ) (left panels) or MAF-specific siRNA-transfected (siRNA MAF M-MØ) (right panels) versus the transcriptome of control siRNA-transfected macrophages (siRNA control M-MØ). Normalized Enrichment Score (NES) and FDRq values of each analysis are indicated.

To gather additional support for the hypothetic role of MAFB and MAF in COVID-19 pathogenesis, we integrated our gene expression data for MAFB and MAF within a “COVID-19 disease framework” built from data derived from single cell transcriptomic experiments on macrophage populations isolated from the lungs of both healthy and COVID-19 patients (29). Three main macrophage populations have been identified in the interstitial space and bronchoalveolar lavage fluid (BALF) from normal lungs samples (30), namely, FCN1^high^, SPP1^high^ and FABP4^high^. Analysis of bronchoalveolar lavage fluid (BALF) from COVID-19 patients has revealed a markedly increased proportion of FCN1^high^ and SPP1^high^ macrophage subsets in lungs from severe COVID-19 patients, what correlates with disease progression, while the proportion of the resident-like Alveolar Macrophages (AM) FABP4^high^ subset is diminished (29) (**Figure 3A**, left panel). The FCN1^high^ subset is derived from circulating monocytes, while the origin of the SPP1^high^ (MERTK^high^) subset is so far unclear (pulmonary or monocyte-derived) (30–32). Whereas the FCN1^high^ subset displays a pro-inflammatory phenotype (29), the SPP1^high^ subset is increased in pulmonary fibrosis (30), is located in fibrotic areas (30, 31), and its gene signature and co-localization with fibroblast foci strongly suggests their identity as the key profibrotic macrophage in human pulmonary fibrosis (32–35). Importantly, self-maintenance and persistence of the SPP1^high^ subset is dependent on M-CSF/M-CSFR signaling (35), what correlates with their specific expression of MAFB (33, 34), the high number of MAFB+ CD68+ macrophages detected in pulmonary fibrosis patients (33, 34) and their expression of MAFB-dependent pro-fibrotic genes like *LGMN* (16, 36, 37). Finally, the FABP4^high^ subset represents the GM-CSF–dependent resident alveolar macrophages, which are essential for the maintenance of lung homeostasis.

To assess the pathological significance of our hypothesis, the expression of the genes most differentially expressed by the FCN1^high^, SPP1^high^, and FABP4^high^ subsets (29) was determined in human M-CSF–conditioned macrophages after knockdown of either MAFB or MAF. For MAFB, GSEA revealed that the transcriptome of the pro-fibrotic SPP1^high^ subset is positively enriched in MAFB-dependent genes [groups 2 and 3 in (29), **Figure 3B**, right panel], including genes coding for chemokines that are integral part of the “cytokine storm” (*CCL2*, *CCL3*, *CCL4*, *CCL7*, *CXCL9*, *CXCL10*, *CXCL11*). Conversely, MAFB downregulation resulted in enhanced expression of the genes that characterize the transcriptome of FABP4^high^ alveolar macrophages [AM, group 4 in (29)]. Thus, MAFB appears to shape the transcriptome of the pathogenic pro-fibrotic SPP1^high^ macrophage subset, whose levels are increased in severe COVID-19 (16, 29). This finding is also in agreement with the MAFB-dependent expression of numerous genes involved in fibrosis, including *LGMN*, *THBS1*, *FBLN5*, *GAS6*, *SERPINB2*, *PRLR*, and *FBN2* [(16) and GSE155719].

In the case of MAF, GSEA revealed that the gene signature of the FCN1^high^ subset [designated as groups 1 and 2 in (29)], is enriched in MAF-inhibited genes, as the Groups of genes preferentially expressed by the FCN1^high^ subset (Group 1 and 2) include a significant over-representation of genes upregulated upon MAF knock-down (**Figure 3B**, right panel). Of note, the specific transcriptome of the FCN1^high^ subset includes genes coding for chemokines (e.g., *CCL2*, *CCL3*, and *CXCL10*) and pro-inflammatory factors, in agreement with its “classic M1-like macrophage” phenotype (29) (**Figure 3B**, right panel). Conversely, and unlike MAFB, MAF expression appeared to have an opposite effect on the expression of genes specifically associated to the FABP4^high^ subset [group 4 in (29)]. Therefore, these data imply that MAF acts as a brake for the pro-inflammatory macrophage activation, and suggest that deprivation of MAF expression also contributes to shaping the transcriptional profile of pro-inflammatory lung macrophages with a pathogenic role in COVID-19. Moreover, analysis of the leading edge genes in their respective GSEA contours showed that MAFB and MAF oppositely regulate the expression of chemokines that contribute to the SARS-CoV-2–induced “cytokine storm” (*CCL2*, *CCL4*, *CCL7*, *CCL8*, *CXCL10*, *CXCL11*), some of which have been already proposed as useful biomarkers for COVID-19 severity and progression (38). Consequently, we hypothesize that MAFB and MAF shape the transcriptome of the fibrotic SPP1^high^ and inflammatory monocyte-derived FCN1^high^ pulmonary macrophage subsets, respectively, which exert a pathogenic role in severe COVID-19. If so, the expression of genes oppositely regulated by MAFB and MAF might constitute helpful biomarkers for COVID-19 severity, as already demonstrated in the case of CXCL10 (38), and imply that the MAFB/MAF expression ratio in lung macrophages is a critical determinant for COVID-19 severity and progression.

Further supporting our hypothesis, additional GSEA also revealed co-enrichment of genes positively regulated by MAFB and negatively regulated by MAF in bronchoalveolar lavage fluid cells in COVID-19 patients (39) (data not shown). In fact, a similar result was observed in other pathological and experimental settings, including synovial membranes from Rheumatoid Arthritis patients [GSE1919 (40)], as well as LPS-activated Alveolar Macrophages [GSE40885 (41)] and IFNα-activated monocyte-derived macrophages [GSE16755 (42)] (data not shown). More importantly, analysis of the DisGeNET (https://www.disgenet.org) and Disease Ontology (DO, https://disease-ontology.org) databases of gene-disease associations also disclosed that the genes positively regulated by MAFB and negatively regulated by MAF are significantly associated to infectious/inflammatory diseases (e.g., pneumonia, glomerulonephritis, juvenile arthritis, rheumatoid arthritis, multiple sclerosis,…) (data not shown). Taken together, these data provide an additional support for the occurrence of an altered effective MAFB/MAF ratio in severe COVID-19 and chronic inflammatory disorders.

To deepen into the potential molecular mechanisms underlying the control of gene expression by a high MAFB/MAF expression ratio in macrophages, we searched for transcription factors differentially modulated upon knock-down of MAFB or MAF in M-CSF–derived macrophages (GSE155719). This analysis revealed that the *Aryl hydrocarbon Receptor* (AhR) is positively regulated by MAFB while negatively regulated by MAF. AhR is a ligand-activated transcription factor that regulates inflammatory responses, shapes adaptive immunity and controls the differentiation potential of monocytes (43). In this scenario, MAFB and MAF differentially affect the expression of *AHR*, and an enhanced MAFB/MAF ratio correlates with an increased expression of *AHR*, as it is also seen in macrophages activated by TLR4 or TLR7 ligands (our transcriptional information in GSE156921). The MAFB/MAF ratio-*AHR* expression link is of special relevance because AhR is activated during coronavirus infection and the constitutive AhR activation constrains type I IFN-mediated antiviral innate defense (44, 45). In fact, a role for AhR in SARS-CoV-2 pathology has been recently proposed (46), and AhR antagonists have been propositioned as potential therapy for coronavirus-infected patients (46). Therefore, considering the link between MAFB/MAF ratio and *AHR* expression, the proposed involvement of AhR in COVID-19 pathogenesis is well in agreement with our hypothesis on the contribution of MAFB and MAF to severe COVID-19.

## Discussion

The altered MAFB/MAF expression ratio that we have found might have prognostic value in COVID-19 severity and progression, as well as potential therapeutic implications. On the prognosis issue, an immediate consequence of our hypothesis would be that the genes positively regulated by MAFB or negatively regulated by MAF could be prognostic biomarkers for severe COVID-19. In this regard, recent reports seem to support the validity of our hypothesis and its prognostic significance. In fact, some of the genes whose expression is oppositely regulated by MAF and MAFB have been already described as biomarkers for COVID-19 severity, including CXCL10 (38) and, more recently, CCL19 (47). Moreover, SARS-CoV-2 infected individuals have a consistent chemokine signature that appears to be a driving feature of COVID-19 infection and includes monocyte-associated chemokines like CCL2 and CCL8 (48), whose expression is also differentially regulated by MAF and MAFB (*Simón-Fuentes et al.*, *manuscript in preparation)*.

Regarding the potential therapeutic value of altering the MAFB/MAF ratio, it is worth noting that both factors are similarly regulated by GSK3β (8), and that the effect of the MAF inhibitor Nivalenol (19) on MAFB is currently unknown. Therefore, and considering their different capacity to heterodimerize with several AP-1 superfamily factors (8), it is tempting to postulate that the heterodimeric partners of MAFB and MAF might represent points of intervention to modulate the effective MAFB/MAF ratio. Initial experiments on this issue indicate that JNK inhibition preferentially impairs the expression of MAF and MAF-regulated genes without affecting the expression of MAFB-dependent genes (*Simón-Fuentes et al.*, *manuscript in preparation*), illustrating the feasibility of modulating the effective MAFB/MAF ratio in human macrophages through JNK inhibition. Thus, although MAPK inhibitors would affect numerous intracellular signaling pathways and cellular functions, this result poses the question of whether available modulators of JNK and MAPK, already used in numerous clinical trials (49) might be useful alternatives for reversing the outcome of the prevailing MAFB/MAF ratio in lung macrophages in COVID-19.

On the other hand, the possibility of altering the MAFB/MAF ratio as a potential therapy for severe COVID-19 raises the question of the appropriate timing for such an approach. The issue of the more adequate time for therapeutic modulation of the inflammatory response in COVID-19 is a matter of debate and is very dependent on the course of the disease. As an example, strategies aim at targeting the GM-CSF/GM-CSF receptor axis have been proposed for distinct stages of the disease (50). In the case of MAFB/MAF, and in spite of the fact that both factors regulate the expression of monocyte-recruiting chemokines and that monocytes give rise to the pathogenic pulmonary macrophages in COVID-19, we envision that altering the MAFB/MAF ratio should only be tried during the overwhelming pro-inflammatory response at the later stages of the disease, a time at which the consequences of altering myeloid differentiation would be less detrimental.

The link between the MAFB/MAF ratio and the gene profile of pathogenic macrophages in severe COVID-19 has also mechanistic implications. In this respect, it is reasonable to assume that the influence of MAF and MAFB on SARS-CoV-2–induced inflammation and fibrosis not only reflects their specific transcriptional activities, but also their capacity to heterodimerize with members of the “large MAF” family as well as members of the AP-1 superfamily of transcription factors (8). In this regard, MAFB can dimerize with JUN, FOS and FRA1/2, while MAF dimerization partners include FOS and ATF4 (8). Notably, AP-1 superfamily factors are major effectors of MAPKs, whose activity modulates the macrophage inflammatory program elicited by PAMP receptors (51, 52) and is connected to the occurrence of the “cytokine storm” during viral responses (53–55). Therefore, it is conceivable that alterations in the MAFB/MAF ratio in macrophages may indirectly dysregulate the modulatory action of MAPKs on pro-inflammatory cytokine/chemokine expression by shifting the transcriptional functions of AP-1 factors. If that is the case, the currently available MAPK-modifying drugs might represent potential therapeutic alternatives for severe COVID-19.

**Figure 4:**
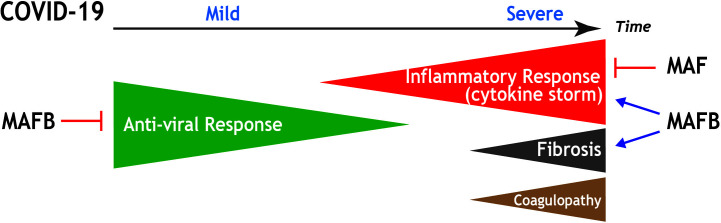
Summary of the potential contribution of MAF and MAFB to progression and main pathological features in COVID-19. Schematic representation of the stages of increasing severity of COVID-19. The anti-viral response prevails at the early infection, in which upper respiratory tract infection predominates (Mild COVID-19). As the disease progresses, the hyperinflammation phase ensues, in which patients develop acute respiratory distress syndrome, sepsis, and organ failures (Severe COVID-19). Severe COVID-19 is characterized by an exacerbated inflammatory response (cytokine storm), as well as by the appearance of pulmonary fibrosis and coagulopathies. Based on functional and transcriptional information, the positive or negative influence of MAF and MAFB in the distinct processes is indicated.

In conclusion, and based on currently available transcriptional information, we hypothesize that MAFB and MAF transcription factors are key players in the pathogenic response of lung macrophages to SARS-CoV-2 infection. In the case of MAFB, its function as a “rheostat” for type I IFN production, and its ability to shape the transcriptome of pro-fibrotic SPP1^high^ lung macrophages, suggest its participation in the defective type I IFN production and the pro-fibrotic response that characterize severe COVID-19. Regarding MAF, its ability to limit the expression of pro-inflammatory cytokines and chemokines, and its negative effect on the acquisition of the transcriptome of FCN1^high^ pulmonary macrophages, suggests its involvement in triggering the pro-inflammatory “cytokine storm”, a major trademark of COVID-19. The association of the expression of MAFB with the expression of a significant number of monocyte-recruiting chemokines induces us to propose that MAFB also contributes, although possibly with a lower extent than MAF, to the “cytokine storm”. Thus, we propose that the expression levels of MAFB and MAF in FCN1^high^ and SPP1^high^ pulmonary macrophage subsets are critical determinants for the defective production of IFN type I, the overwhelming hyper-inflammatory response, and the exacerbated profibrotic response that takes place upon severe SARS-CoV-2 infection (**Figure 4**). By extension, MAFB/MAF expression ratio in lung macrophages appears to be a critical determinant for COVID-19 severity and progression and, therefore, therapies directed to simultaneously silencing MAFB and overexpressing MAF in pulmonary macrophages might constitute suitable strategies to combat COVID-19.

## Data Availability Statement

The datasets presented in this study can be found in online repositories. The names of the repository/repositories and accession number(s) can be found at https://www.ncbi.nlm.nih.gov/(GSE155719) and https://www.ncbi.nlm.nih.gov/(GSE156921).

## Ethics Statement

Ethical approvals for all blood sources and processes used in this study were approved by the Centro de Investigaciones Biológicas Ethics Committee. The ethics committee waived the requirement of written informed consent for participation.

## Author Contributions

MS-F, AG, CN, MC, CH, and ÁD-S performed research and analyzed data. ÁD-S, MV, and AC designed the research and analyzed data. MV and AC wrote the paper. All authors contributed to the article and approved the submitted version.

## Funding

This work was supported by grants from Consejo Superior de Investigaciónes Científicas (202020E228), Ministerio de Economía y Competitividad (SAF2017-83785-R), and AYUDAS FUNDACIÓN BBVA A EQUIPOS DE INVESTIGACIÓN CIENTÍFICA SARS-CoV-2 y COVID-19 to MV and AC, Grant 201619.31 from Fundación La Marató/TV3 to AC, and Red de Investigación en Enfermedades Reumáticas (RIER, RD16/0012/0007) from Instituto de Salud Carlos III and cofinanced by the European Regional Development Fund “A way to achieve Europe” (ERDF). We acknowledge support of the publication fee by the CSIC Open Access Publication Support Initiative through its Unit of Information Resources for Research (URICI). We thank José Luis Rodríguez, Antonio Castrillo, and Silvia Sánchez Ramón for suggestions and critical reading of the manuscript.

## Conflict of Interest

The authors declare that the research was conducted in the absence of any commercial or financial relationships that could be construed as a potential conflict of interest.
